# Relationship between physical parameters and visual analysis for assessment of image quality: a multi-center and multi-vendor phantom study in brain SPECT

**DOI:** 10.22038/aojnmb.2024.75204.1524

**Published:** 2025

**Authors:** Akie Sugiura, Takayuki Shibutani, Masahisa Onoguchi, Akio Nagaki, Kotatsu Tsuboi, Toshimune Ito, Hajime Ichikawa

**Affiliations:** 1 Department of Quantum Medical Technology, Graduate School of Medical Sciences, Kanazawa University,; 2 Kanazawa, Ishikawa, Japan; 3 Department of Radiological Technology, Kurashiki Central Hospital, Okayama, Japan; 4 Department of Radiological Technology, Hamamatsu Red Cross Hospital, Shizuoka, Japan; 5 Department of Radiological Technology, Faculty of Medical Technology, Tokyo, Japan; 6 Department of Radiology, Toyohashi Municipal Hospital, Aichi, Japan

**Keywords:** Brain perfusion SPECT, Multi-center, Multi-vendor, Physical parameter, Visual analysis

## Abstract

**Objective(s):**

Brain perfusion single-photon emission computed tomography (SPECT) image quality varies depending on SPECT systems. This study aimed to evaluate the relationship between physical parameters and visual analysis for assessment of the brain SPECT image quality. We conducted our phantom study under various conditions in a multi-center and multi-vendor study.

**Methods:**

SPECT images of the brain phantom were acquired from eight devices in five institutions. The phantom was filled with 28 kBq/ml of ^99m^Tc solution at the start of scanning. We obtained various data with different acquisition times under clinical reconstruction and acquisition conditions at each institution. Four physical parameters (percent contrast, contrast noise ratio (CNR), asymmetry index (AI), and sharpness index (SI)) were measured with the phantom. Seven observers blindly evaluated all image series and scored them on a scale of 1–3 using four checkpoints: contrast, image noise, symmetry, and sharpness. The average score for all observers was calculated.

**Results:**

CNR increased with increasing visual analysis scores for contrast and image noise, both of which were significantly different between the group with scores “<2” and the group with scores “≥2 and <3”. AI decreased as the visual analysis score for symmetry increased, and the AI of both groups with scores “≥2 and <3” and “3” were significantly lower than that of the group with scores “<2”. Conversely, no relationship with visual analysis was found for percent contrast and SI.

**Conclusion:**

We clarified the relationship between physical parameters and visual analysis of a brain phantom in a multi-center and multi-vendor study. CNR and AI showed agreement with visual analysis.

## Introduction

 Brain perfusion single-photon emission computed tomography (SPECT) imaging has been widely used to measure regional cerebral perfusion and is commonly indicated for the assessment of ischemic and hyperperfused areas with cerebrovascular diseases and differential diagnosis of dementia (1). It also provides functional information on the perfusion and metabolic status of brain tissue, which cannot be obtained by structural neuroimaging, such as computed tomography (CT) and magnetic resonance imaging. SPECT plays a crucial role in the diagnosis, therapeutic management, and follow-up of patients with cerebral disease because functional impairment often precedes structural changes (2). In addition, SPECT is a useful tool for research because it can monitor human brain function noninvasively (3). 

 However, SPECT image quality depends on various SPECT systems, including a collimator or detector trajectory. Furthermore, image quality is also influenced by the reconstruction parameters even with similar radioactivity distribution (4). Recently, the development of corrections for photon attenuation and scatter, collimator modeling, and three-dimensional (3D) reconstruction has significantly improved image quality; however, these improvements have increased inter-center and inter-vendor variability (5-9) and recent studies have dealt with optimization and standardization in brain perfusion SPECT (10-13). In addition, recent developments on SPECT hardware and software are expected to further stimulate discussions on image quality assessment in relation to imaging techniques with high resolution, high sensitivity, and short acquisition time.

 However, no studies have reported in detail the relationship between physical parameters and visual analysis for assessment of image quality, across different centers or vendors. Physical parameters allow objective and reproducible assessment of image quality and have been commonly used for the optimization and standardization approach of image quality (10-14). However, imaging diagnosis ultimately relies on a visual interpretation by radiologists. 

 Therefore, it is important to establish the relationship between physical parameters and visual analysis. Establishing of the relationship between physical parameters and visual analysis among different centers or vendors will also be helpful to obtain reliable data in multi-center studies or optimize parameters for SPECT system upgrades and renewals.

 Therefore, the goal of this study was to evaluate the relationship between physical parameters and visual analysis by selecting several physical parameters measured from a brain phantom under various conditions in a multi-center and multi-vendor study. As SPECT hardware and software continue to evolve, gain in complexity and diverse, multi-center and multi-vendor studies such as this one will be necessary to ensure that brain perfusion SPECT remains a universal tool for research, clinical trials, and patient management.

## Methods

***Phantom design***

 We chose the Hoffman 3D brain (Data Spectrum Corporation, Durhum, United states) to simulate gray- and white-matter structures with 4:1 radioactivity concentration (15). The same phantom was scanned in all institutions. The phantom was filled with 28 kBq/ml of ^99m^Tc solution at the start of scanning. This is assumed to be the normal accumulation of ^99m^Tc-ethyl-cysteinate dimer for brain uptake of 5.5 % (16). The dose was determined at twice the dosage of a previous study (10) to set the acquisition time for half the target time and conduct experiments efficiently.

***Image acquisition and image reconstruction***

**Figure 1:**
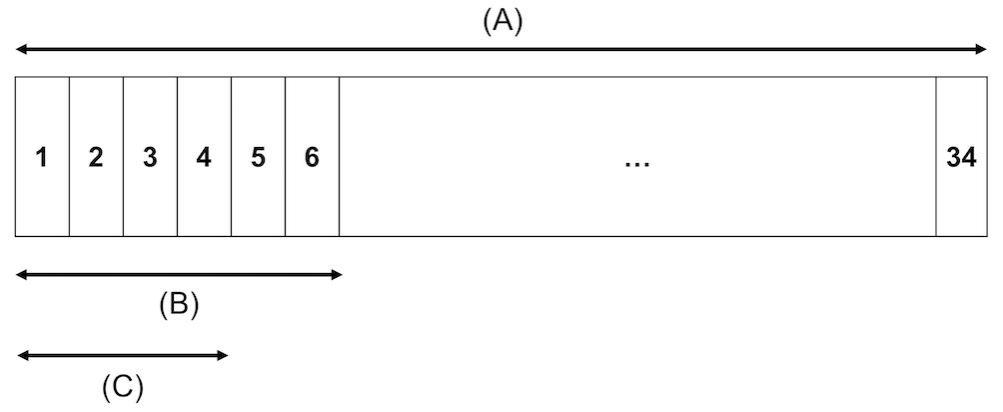
An example of a SPECT acquisition protocol used in this study. SPECT data were acquired for an hour (150 s 34 rotations) (**a**) Clinical acquisition time (150 s 6 rotations) (**b**) Short acquisition time (150 s 4 rotations) (**c**)

 SPECT images of the Hoffman 3D brain phantom were acquired from eight SPECT or SPECT/CT devices in five institutions, and SPECT data were obtained in repetitive rotations. The acquisition time was set to obtain enough acquisition counts equal to two hours (12). In addition, we obtained various data with different acquisition times, such as the clinical condition and short time, by dividing the hour data. Figure 1 illustrates an example of a SPECT acquisition protocol used in this study. Table 1 shows the acquisition conditions, including scanner, collimator, and acquisition time for each device. SPECT images were reconstructed with clinical parameters at each institution. Table 2 shows the reconstruction conditions that were used in this study.

**Tabel 1:** Acquisition conditions used in multicenter studies

Laboratory*	**Vendor, model**	**Collimator** ^†^	**Radius (mm)**	**Matrix**	**Zoom**	**Pixel size (mm)**	**Acquisiton times (sec/rotation)**	**Acquisition mode**	**Step angle (degree)**	**Energy window**
Kariya	Discovery NM/CT 670 Q.Suite pro, GE Healthcare Japan	LEHR	150	128 × 128	1.5	2.94	105 (2, 3, 4, 5, 6†,34 rotation)	continuous	4, circular orbit	140.5 keV ± 10 % (sub-windows; 159.5 keV ± 3 %, 121.5 keV ± 4 %)
Kanazawa	Discovery NM/CT Q.Suite pro, GE Healthcare Japan	LEHRS	150	128 × 128	2	2.21	75 (2, 4, 6, 8†,48 rotation)	continuous	4, circular orbit	140.5 keV ± 10 % (sub-window; 120 keV ± 5 %)
Kanazawa	Discovery NM/CT Q.Suite pro, GE Healthcare Japan	LEHR	150	128 × 128	2	2.21	75 (2, 4, 6, 8†, 48 rotation)	continuous	4, circular orbit	140.5 keV ± 10 % (sub-window; 120 keV ± 5 %)
Kanazawa	GCA9300R, Canon Medical Systems Corporation	FANHR	132	128 × 128	1	3.2	75 (2, 4, 6, 8†, 48 rotation)	continuous	4, circular orbit	141 keV ± 20 % (sub-window; 7 %)
Kanazawa	Sinbia Intevo16, Siemens Healthineers	LEHR	150	128 × 128	2.29	2.1	75 (2, 4, 6, 8†, 48 rotation)	continuous	4, circular orbit	140 keV ± 10 % (sub-window; 7 (%)
Yokohama	ECAM.signature, Canon Medical Systems Corporation	LEHR	140	128 × 128	1.45	3.3	75 (2, 4, 6, 8,10†, 48 rotation)	continuous	4, circular orbit	140 keV ± 10 % (sub-window; 7 (%)
Kurashiki	Sinbia T2, Siemens Healthineers	LEHR	140	128 × 128	1.45	3.3	60 (2, 4, 6, 8,10†, 60 rotation)	continuous	3, circular orbit	125.3-154.7 keV (sub-window; 115.5-125.3 keV (Lower))
Hamamatsu	ECAM.signature, Canon Medical Systems Corporation	LEHR	140	128 × 128	1.45	3.3	75 (1, 2, 3, 4, 5, 6^$^, 48 rotation)	continuous	4, circular orbit	140 keV ± 7.5 % (sub-window; -(%)

* **Laboratory:** Kariya, Kariya toyota general hospital; Kanazawa, Kanazawa University Hospital; Yokohama, Saiseikai Yokohamashi Tobu hospital; Kurashiki,Kurashiki Central hospital; Hamamatsu,Japanese Red Cross Hamamatsu Hospital

**†** **Collimator: **LEHR, low-energy high resolution; LEHRS, low-energy high resolution sesitivity; FANHR, fan beam high resolution

**$: **Acquision parameters in clinical studies

**Tabel 2:** Reconstruction conditions used in multicenter studies

**Laboratory**	**Vendor, model**	Reconstruction method*	**Reconstruction filter**	**Iteration/subset**	**Filter name†**	**Filter parameters$**	**Corrections#**	**Slice thickness (mm)**
Kariya	Discovery NM/CT 670 Q.Suite pro, GE Healthcare Japan	FBP	Ramp	-	BW	0.55/ 16 (cycles/cm)	AC (Chang, μ:0.13 cm-1), SC (triple-energy window (TEW) )	5.89
Kanazawa	Discovery NM/CT 670 Q.Suite pro, GE Healthcare Japan	FBP	Ramp	-	BW	0.5/ 10 (cycles/cm)	AC (Chang, μ:0.11 cm-1), SC (dual-energy window (DEW) )	2.21
Kanazawa	Discovery NM/CT 670 Q.Suite pro, GE Healthcare Japan	FBP	Ramp	-	BW	0.5/ 10 (cycles/cm)	AC (Chang, μ:0.11 cm-1), SC (DEW)	2.21
Kanazawa	GCA9300R, Canon Medical Systems Corporation	OSEM	-	10/10	BW	0.64/ 4 (cycles/cm)	AC (Chang, μ:0.1 cm-1), SC (TEW)	1.72
Kanazawa	Sinbia Intevo16, Siemens Healthineers	OSEM	-	12/10	G	10.5 (mm)	RR, AC (computed tomography-based attenuation correction (CTAC) ), SC (TEW)	2.1
Yokohama	ECAM.signature, Canon Medical Systems Corporation	FBP	Ramp	-	BW	0.5/8 (cycles/cm)	AC (Chang, μ:0.15 cm-1), SC (TEW)	3.3
Kurashiki	Sinbia T2, Siemens Healthineers	OSEM	-	6/10	G	8.4 (mm)	RR, AC (CTAC), SC (multiple-energy window (MEW) )	3.3
Hamamatsu	ECAM.signature, Canon Medical Systems Corporation	FBP	Ramp	-	BW	0.61/8 (cycles/cm)	AC (Chang, μ:0.09 cm-1), SC-	3.3

*Reconstriction method: FBP, filtered back projection; OSEM, ordered-subsets expectation maximization

**†** **Filter name:** G, Gaussian; BW, Butterworth

**$** **Filter parameters:** BW (cut-off/order), G (full width at half maximum (FWHM) )

**#**: Corrections: AC, attenuation correction; SC, scatter correction; RR, resolusion recovery

***Physical parameters for assessment of image quality***

 We measured the following four physical parameters measurable in Hoffman 3D brain phantom experiments: percent contrast as a physical parameter for contrast (10, 14), contrast noise ratio (CNR) as a physical parameter for contrast and image noise (13), asymmetry index (AI) as a physical parameter for symmetry (17), and sharpness index (SI) as a physical parameter for sharpness (18).

 % Contrast and CNR: Contrast and image noise were evaluated in the following manner (19). First, the SPECT images from each device were registered to CT images of a device (matrix size, 512×512; pixel size, 0.586×0.586 mm^2^) using Fusion Viewer (Nihon Medi-Physics Co., Ltd., Tokyo, Japan). This step aimed to place regions of interest (ROIs) on the same slice of a Hoffman 3D brain phantom for all images, and a basal ganglia level slice was used as the representative slice for the ROI setting (20). 

 Second, the pixel size of registered CT and SPECT images was linearly interpolated to 1×1 mm^2^ to prevent partial volume effects. 

 Subsequently, to set same-size ROIs to the same location for all images, the following ROI template was defined using an open-source software (ImageJ v1.52, National Institutes of Health, Bethesda, MD): gray-matter ROIs in the representative slice, which was automatically delineated with -5 <HU < 5 thresholds on the CT image, and white-matter ROIs in the representative slice, which was automatically delineated with thresholds of 65 < HU < 85. 

**Figure 2:**
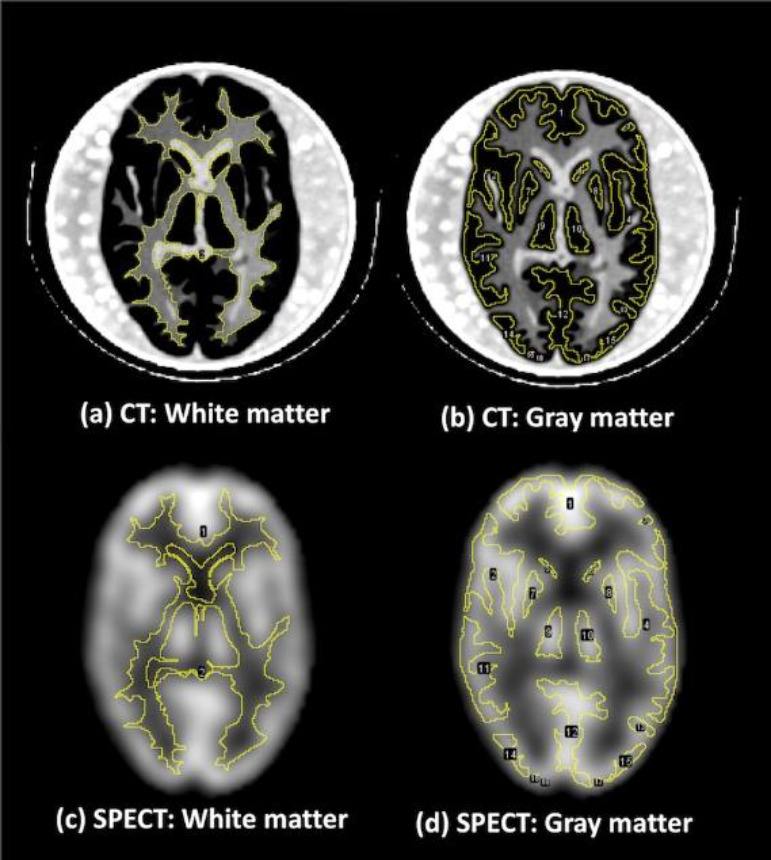
White-matter ROIs on the registered CT image (**a**) Gray-matter ROIs on the registered CT image (**b**) White-matter ROIs on the registered SPECT image (**c**) Gray-matter ROIs on the registered SPECT image (**d**)

 Small delineation errors were corrected manually. The ROI template obtained was set to SPECT images (Figure 2). The percent contrast and CNR were calculated as follows (10, 13-14):

$\mathrm{\%contrast}=\frac{\mathrm{GM}/\mathrm{WM}}{{\mathrm{GM}}_{\mathrm{ref}}/{\mathrm{WM}}_{\mathrm{ref}}}\times 100[\%]$

$\mathrm{CNR}=\frac{\mathrm{GM}-\mathrm{WM}}{{\mathrm{SD}}_{\mathrm{WM}}}$

 Where GM and WM were the averages of reconstructed image counts of gray- and white-matter ROIs measured on each phantom image, respectively. GMref and WMref were the averages of the reconstructed image counts of gray- and white-matter on the reference phantom image, respectively. In this study, the reference image was created by the projection data with high acquisition counts, which were equal to the hour data, and reconstructed using filtered back projection without a filter (10). 

 SD_WM_ was the standard deviation of white matter ROIs measured on each phantom image. 

*AI*: Symmetry was evaluated in the following manner. ROIs of the right and left thalamus were automatically drawn on each phantom image using the 3D stereotaxic ROI template software (PDRadiopharma Inc., Tokyo, Japan) (21). The AI is given using the following equation (17):

$\mathrm{AI}=\frac{\left|{\mathrm{thalamus}}_{\mathrm{rt}}-{\mathrm{thalamus}}_{\mathrm{lt}}\right|}{\left({\mathrm{thalamus}}_{\mathrm{rt}}+{\mathrm{thalamus}}_{\mathrm{lt}}\right)}$

 Where the thalamusrt and thalamuslt were the average reconstructed image counts of the right and left thalamus ROIs measured on each phantom image, respectively. 

**Figure 3:**
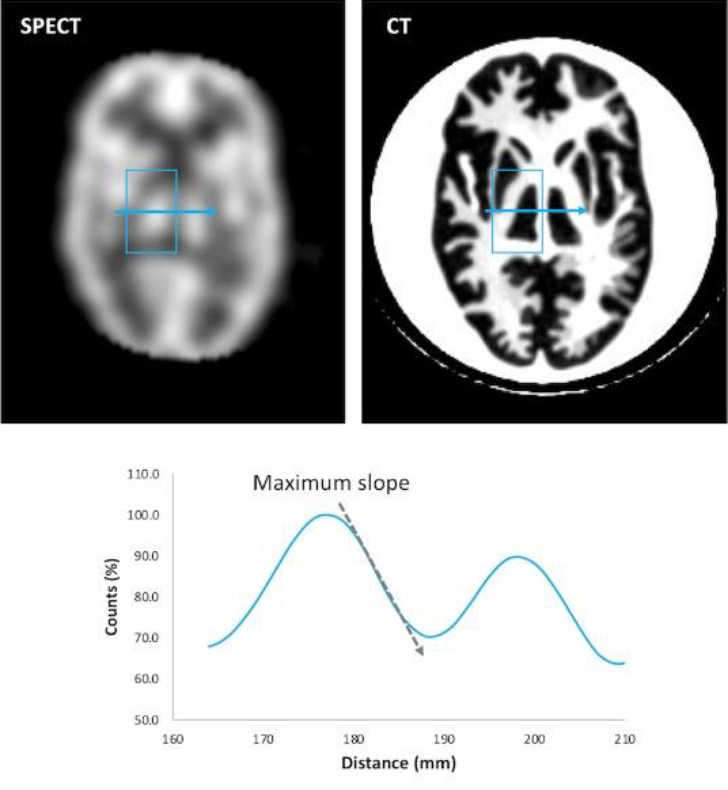
The maximum slope of count profiles for the sharpness index. The count profile was placed across the right thalamus. Distance is expressed in millimeters, and counts are expressed in the percentage of maximum pixel counts

*SI*: As illustrated in Figure 3, sharpness was assessed using the SI defined on the count profile. The count profile was placed across the right thalamus on the aforementioned representative slice of CT images. This index was calculated as the maximum slope of decrease counts (mm^-1^) on the gray- and white-matter border of the right thalamus and expressed as a percentage of the maximum count values and per millimeter length (18, 22).

***Visual analysis***

**Figure 4:**
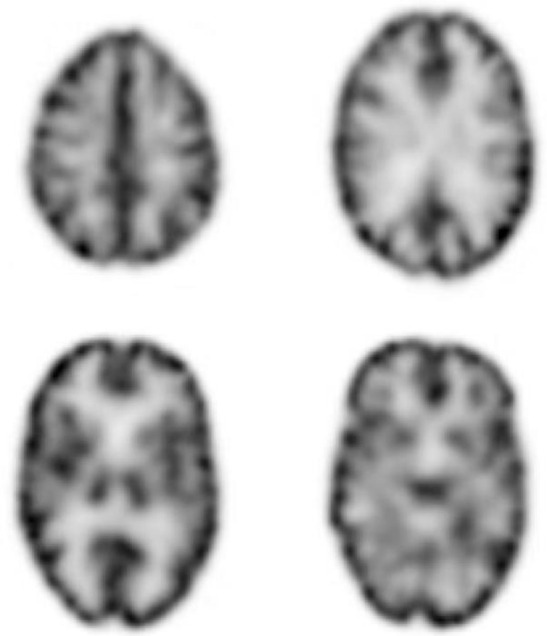
The representative slice images of a Hoffman 3D brain phantom for visual analysis

 Forty-five types of series were sent to seven experts (two physicians with over 5 years experiences in reading brain perfusion SPECT images and five board certified nuclear medicine technologists in charge of nuclear medicine examinations) who were familiar with the precise structures of the phantom based on the CT images. They made assessments via visual analysis. We also selected four slices as the representative slice images of the Hoffman 3D brain phantom (Figure 4) (20). To make visual analysis more objective, four checkpoints were evaluated: contrast, image noise, symmetry, and sharpness. The details are as follows.

***Contrast:*** We evaluated whether the contrast between the gray and white matter was enough.

***Image noise:*** We evaluated whether cortical accumulation was properly smoothed.

***Symmetry:*** We evaluated whether the thalamus was symmetrical and homogenous.

***Sharpness:*** We evaluated whether the counter for the thalamus was clear.

 Observers blindly evaluated the image series and scored them on a scale of 1–3 using four checkpoints. Details of the scores are as follows: 1, poor; 2, mediocre; and 3, good. The color lookup table was set to inverted grayscale. Furthermore, the enlargement and reduction of the images, observation distance, and observation time were arbitrary. The average score for all observers was calculated and evaluated based on the corresponding physical parameters.

***Statistical analysis***

 Statistical analysis was performed with Kruskal-Wallis/Steel’s test using the statistical package EZR (ver.1.38) (23). For all analyses, a probability (p) value of <0.05 and 0.01 was considered statistically significant.

## Results

**Figure 5:**
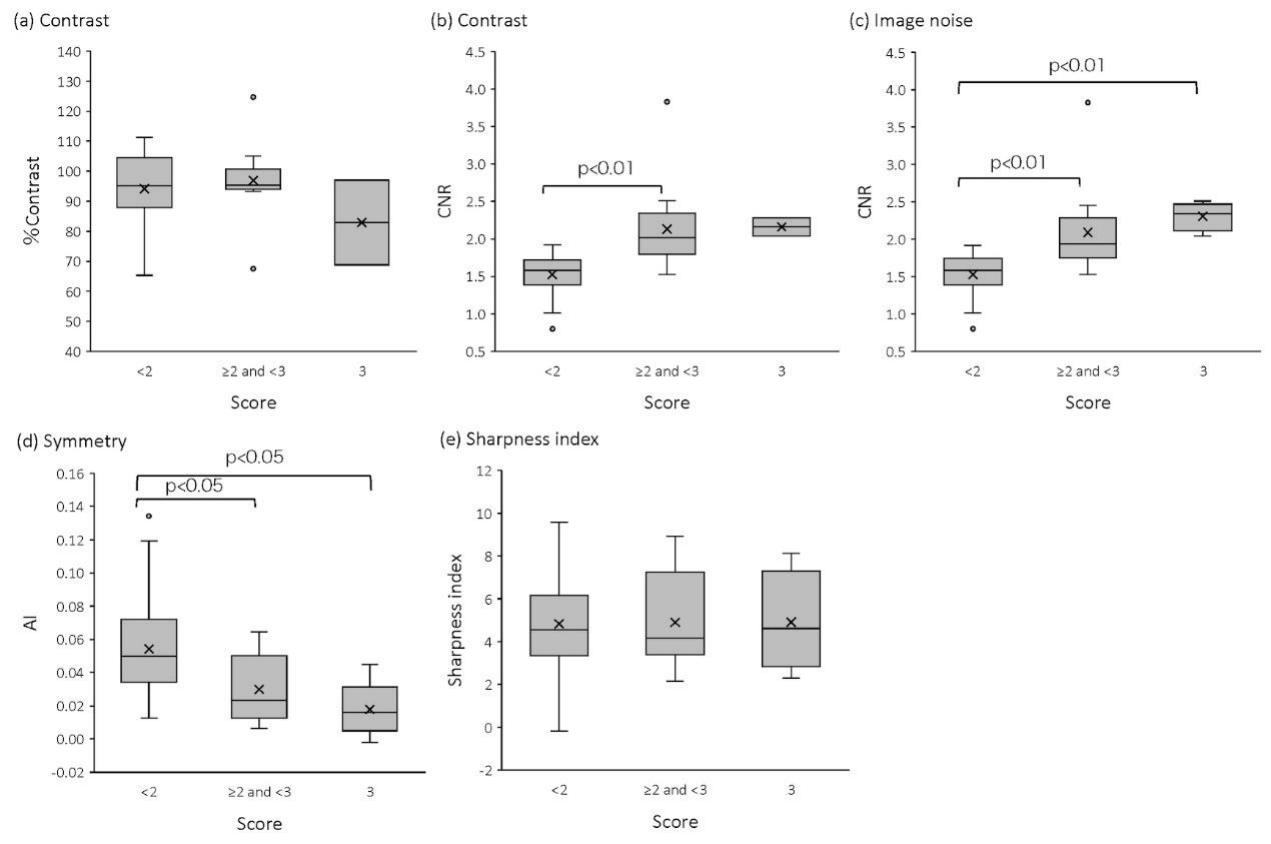
Box plots of percent contrast (**a**), CNR (**b**, **c**), AI (**d**), and sharpness index (**e**) at visual analysis scores (contrast, image noise, symmetry, and sharpness) of <2, ≥2 or <3, and 3

Table 3 summarized the relationship between four physical parameters and visual analysis scores. Figure 5a shows the relationship between percent contrast and visual analysis scores for contrast. The average values of percent contrast were 94.2±13.1 (<2), 96.9±9.9 (≥2 and <3), and 82.9±20.0 (3), and no significant differences were observed among the three groups. Figure 5b shows the relationship between CNR and visual analysis scores for contrast. The average values of CNR were 1.5±0.3 (< 2), 2.1±0.5 (≥2 and <3), and 2.2±0.2 (3), and a significant difference was observed between the group with scores “<2” and the group with scores “≥2 and <3” (p< 0.01). 

 No significant differences were observed between the group with scores "<2" and that with scores of "3" (p=0.06), and between the group with scores “≥2 and <3” and that with scores of “3” (p=0.94). However, increasing trends were observed. Figure 5c shows the relationship between CNR and visual analysis scores for image noise. The average values of CNR were 1.5 (< 2), 2.1 (≥2 and <3), and 2.3 (3), and the CNR averages of both groups with scores “≥2 and <3” and “3” significantly increased, compare to the group with scores “<2”. (all p < 0.01). Although there were no significant differences between the group with scores “≥2 and <3” and the group with scores “3” (p=0.16), an increasing trend was observed. 

Figure 5d shows the relationship between AI and visual analysis scores for symmetry. The average values of AI were 0.05±0.03 (< 2), 0.03 ± 0.02 (≥2 and <3), and 0.02±0.02 (3), and the AI averages of both groups with scores “≥2 and <3” and "3" were significantly lower than that of the group with scores “<2” (all p < 0.05). Although there were no significant differences between the group with scores “≥2 and <3” and that with scores of “3” (p=0.50), a decreasing trend was observed. Finally, SI values were 4.8±2.7 (< 2), 4.9±2.2 (≥2 and <3), and 4.9±2.4 (3), and no significant differences were observed among the three groups (Figure 5e). 

**Figure 6:**
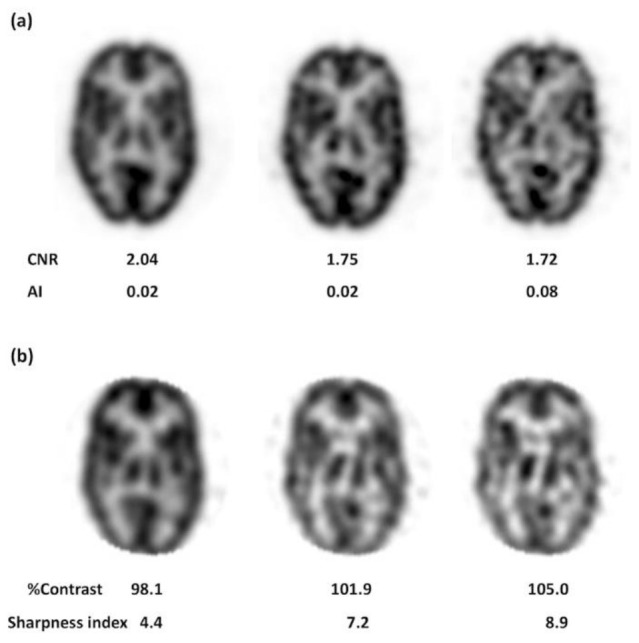
Example images and measured physical parameter values of a brain phantom. (**a**) CNR and AI, (**b**) percent contrast and sharpness index

 In the example shown in Figure. 6a, we observe that cortical accumulation becomes noisy and the contrast between gray and white matter disappears as the CNR values decrease. 

 Furthermore, thalamus symmetry disappears as the AI values increase. However, Figure 6b confirms that the images become noisier and degraded as the percent contrast and SI values increase.

**Table3:** Relationship between four physical parameters and visual analysis scores

**Physical parameter**	**Contrast score**	**Image noise score**	**Symmetry score**	**Sharpness score**
Percent contrast				
CNR	*	*		
AI			*	
SI				

*: Parameters that agreement with visual analysis. These are the parameters that changed significantly with changes in visual analysis scores

## Discussion

 We evaluated the relationship between the physical parameters of a brain phantom and visual analysis in a multi-center and multi-vendor study. Various physical parameters have been used in previous studies of brain SPECT (10, 13-14, 17-18). However, there are no studies that have reported in detail the relationship between physical parameters and visual analysis, across different centers or vendors. To our best knowledge, this is the first time the relationship is addressed.

 We found that CNR and AI showed agreements with visual analysis. The physical parameters were selected based on previous studies (10, 13-14, 17-18).These parameters are suitable for use in multi-center studies because they can be calculated with simple ROIs and profile curve settings and do not require a special environment. In addition, the parameters reflect important factors in determining image quality of brain SPECT, such as contrast, sharpness, image noise, and symmetry.

 Contrast is important to distinguish normal from abnormal regions, and sharpness is essential to accurately determine the anatomical location in brain perfusion SPECT. 

 Image noise degrades image quality. Furthermore, uniformity and symmetry are important in addition to contrast, sharpness, and image noise because perfusion distributions have been associated with types of dementia. Based on these considerations and previous studies, we selected four parameters: percent contrast, CNR, AI, and SI. Although some studies used similar parameters (19, 24), we believe that their results had the same tendencies as ours because they used from ROIs or profile curve settings and simple calculations, such as subtraction or ratio, as well as the four parameters we selected. 

 Percent Contrast: No relationship or trend was found between percent contrast and visual analysis. For example, some of the percent contrast values exceeded 100%, or were higher in low count images than in high count images for some devices. In this study, differences in pixel counts between gray and white matter sometimes increased because of noise. As a result, the gray-to-white matter ratio in the numerator occasionally became greater than that in the denominator. Therefore, visual analysis did not always agree with changes in percent contrast. Although percent contrast is a widely used physical parameter (10, 14), this study suggests that it should be measured under low noise conditions.

 CNR: CNR values increased according to visual analysis scores of contrast and image noise, which were significantly different between the group with scores “<2” and the group with scores “≥2 and <3” for both of contrast and image noise. However, no significant differences were observed between the group with scores “≥2 and <3” and the group with scores “3” for both of contrast and image noise. 

 Furthermore, no significant differences were found between the group with scores “<2” and the group with scores “3” for contrast. As the reason for this, we believe that visual analysis scores plateau at “3” and a sample size of “good” images is small. An additional score of ">3" and a larger sample size of “good” images need to be studied in the future. In this study, we confirmed that CNR was useful in discriminating poor and mediocre or more images. Such usefulness of CNR will be helpful for setting standardized cutoff value for image quality.

 AI: AI became lower as the visual analysis score of symmetry increased, and the AI became significantly lower in both groups with scores “≥2 and <3” and “3” than the group with scores “<2”. However, no significant differences were observed between the group with scores “≥2 and <3” and the group with scores “3”.

 Therefore, an additional score “>3” and a larger sample size of “good” images need to be discussed for AI as well as CNR. A previous study assessed hypo perfusion in the thalamus due to thalamus hematoma using AI, and reported that AI in patients with hematoma was larger than the mean+2SD of AI in control participants (17). Our study agrees with the results of this study. Evaluation of relative cerebral blood flow distribution is important in the differential diagnosis of degenerative diseases such as dementia. Thus, uniform images without left-right or anterior-posterior differences are necessary. Previously, uniformity has been evaluated using pool phantoms (10, 14); however, a more accurate evaluation can be expected with the addition of AI. We clarified that AI, as well as CNR, were useful in discriminating poor and mediocre or more images.

 SI: No relationship or trend was found between SI and visual analysis. This could be due to two reasons. First, the difference in pixel counts between gray- and white-matter occasionally increased because of noise, which also increased for percent contrast (Figure 6b). 

 Second, noise may have caused distortion in the profile curves of the thalamus, resulting in poor measurements in some cases. Those factors may have resulted in the deviation between SI and visual analysis. Julian et al. showed that SI reflected the change in spatial resolution between analog and digital positron emission tomography (PET) (18), which deviated from our results. There are two reasons for this deviation. First, their study showed a significant difference only between analog PET with 2-mm voxel size and digital PET with 1-mm voxel size, but not between digital PET with 2-mm voxel size and digital PET with 1-mm voxel size. Therefore, we consider that SI reflects only marked changes in spatial resolution and not slight changes in spatial resolution. Second, this deviation may also be occurred because their study was performed under more constant acquisition and reconstruction conditions than that in our study. Therefore, SI needs to be measured under constant acquisition and reconstruction conditions.

 For some of the parameters, we were able to reveal their relationship with visual analysis. In addition, we clarified points to keep in mind regarding the acquisition and reconstruction conditions when using physical parameters for image qualities. Our findings will be helpful the setting of appropriate experimental conditions, the selection of physical parameters, and the accurate evaluation of data when evaluating image quality using physical parameters such as multi-center studies, SPECT system upgrades or renewals.

 This study has three limitations. First, CNR decreases with a high-pass filter and increases with a low pass filter (25), which may cause discrepancies between CNR and visual analysis. 

 Future studies should assess CNR using more filter conditions to indicate better agreement with visual analysis.

 Second, we created several images with various qualities in this phantom study and evaluated the relationship between physical parameters and visual analysis. The relationship between physical parameters and image quality should be validated using images with known contrast or uniformity, such as digital phantoms. 

 Finally, our study did not provide physical parameters consistent with sharpness for visual analysis. Recently, physical parameters such as the structural similarity index measure or the peak signal-to-noise ratio have been used to evaluate high-resolution images obtained from artificial intelligence imaging techniques, which have been also applied to analyze high-resolution brain PET (26). Although these parameters have challenges regarding setting the appropriate reference image or ease of use, accurate evaluation of sharpness can be expected to perform.

## Conclusion

 We clarified the relationship between physical parameters and visual analysis of a brain phantom in a multi-center and multi-vendor study. CNR and AI showed agreement with visual analysis, indicating their usefulness as physical parameters. We found that percent contrast and SI needs to be measured under specific acquisition and reconstruction conditions. Our findings among vendors and centers will provide reliable data for multi-center studies and for optimization and assessment of image quality.
